# Coexistence of *bla*_NDM–1_ and *bla*_IMP–4_ in an IncHI5 plasmid harbored by carbapenem- resistant *Raoultella ornithinolytica*

**DOI:** 10.3389/fmicb.2026.1744139

**Published:** 2026-04-22

**Authors:** Yuanye Qu, Weiqiang Xiao, Yanmin Chang, Mingyue Sun, Wenjiao Li, Qingxia Xu

**Affiliations:** Department of Clinical Laboratory, The Affiliated Cancer Hospital of Zhengzhou University & Henan Cancer Hospital, Zhengzhou, Henan, China

**Keywords:** *bla*_IMP–4_, *bla*_NDM–1_, carbapenemase, IncHI5 plasmid, *Raoultella ornithinolytica*

## Abstract

**Background:**

*Raoultella ornithinolytica* is an emerging pathogen. This study aimed to characterize the genomic and molecular features of a carbapenem-resistant *R.ornithinolytica* strain co-harboring *bla*_NDM–1_ and *bla*_IMP–4_.

**Methods:**

Strain he2023 was identified and subjected to antimicrobial susceptibility testing using the BD Phoenix-M50 fully automated system. The strain was verified using MALDI-TOF. Carbapenemase genes were confirmed by PCR. Whole-genome sequencing (WGS) was performed to identify resistance genes and plasmid types. Conjugation experiments were conducted to assess transferability, and plasmid stability was evaluated through serial subculturing.

**Results:**

The BD Phoenix-M50 system misidentified strain he2023 as *Klebsiella pneumoniae*, whereas MALDI-TOF and WGS correctly identified it as *R. ornithinolytica*. The strain was resistant to most antimicrobial agents, remaining susceptible only to amikacin,Tigecycline and polymyxin B. The *bla*_NDM–1_ and *bla*_IMP–4_ genes were both located on the plasmid pNDM-IMP, which belongs to the replicon type IncHI5. The *bla*_NDM–1_ was located in the structure of *ISAba125*-*bla*_NDM–1_-*ble*_MBL_-*trpF*-*dsbC*, residing in a region within the remnant of transposon *Tn125*, flanked by two copies of *ISCR1*. The *bla*_IMP–4_ was located downstream of *bla*_NDM–1_ within a novel class 1 integron (*In1965*) carrying the structure *sul1*-*qacE*-*arr-3-ltrA*-*bla*_IMP–4_-*IntI1*. Conjugation and transformation assays showed that the plasmid pNDM-IMP was non-transferable but exhibited high structural stability.

**Conclusion:**

This study is the first to report an IncHI5 plasmid co-harboring *bla*_NDM–1_ and *bla*_IMP–4_ in *R. ornithinolytica*. Non-transferable plasmids may serve as reservoirs or initial carriers for antibiotic resistance genes. Our findings highlights the importance of mobile genetic elements in the formation of multidrug resistance.

## Introduction

*Raoultella* is a genus of Gram-negative, non-motile, encapsulated bacteria within the family Enterobacteriaceae. The species formerly assigned to *Klebsiella* were later reclassified into the genus *Raoultella* on the basis of phylogenetic analyses of 16S rDNA sequences and of genes such as *rpoB*, *gyrA*, and *gyrB* (Drancourt et al., 2001). *Raoultella* species are widely distributed in natural environments (water, soil, and plants) and can be found in clinical settings, where they act as opportunistic pathogens (Seng et al., 2016). *R. ornithinolytica* is the most common pathogenic species within the genus *Raoultella*. It frequently causes healthcare-acquired infections in immunocompromised individuals, including patients with malignancy, those undergoing prolonged hospitalization or surgery, and solid-organ transplant recipients receiving immunosuppressive therapy (Antimicrobial Resistance Collaborators, 2022). Infections caused by *R. ornithinolytica* include urinary tract infections, pulmonary infections, bacteremia, surgical-site infections, biliary tract infections, and, in severe cases, septic shock. Retrospective analyses (Chun et al., 2015; Seng et al., 2016) have reported mortality rates for *R. ornithinolytica* infections of up to 20%, with rates of 34%–44% for bacteremia. *R. ornithinolytica as* an emerging pathogen, has been implicated in an increasing number of human infection cases, particularly with carbapenem-resistant strains.

Carbapenem-resistant Enterobacteriaceae (CRE) infections are a major global public-health problem, associated with high mortality, substantial morbidity and rising healthcare costs (Dang et al., 2020). As in *Klebsiella* species, the principal mechanism of carbapenem resistance in *R. ornithinolytica* is production of carbapenemases. According to the Ambler classification, carbapenemases are grouped into Class A, Class B, and Class D enzymes (Wu et al., 2019; Puljko et al., 2023). Class A enzymes are predominantly represented by KPC carbapenemases, class B metallo-β-lactamases include NDM and IMP enzymes, and class D enzymes are primarily OXA carbapenemases. Coexistence of NDM and IMP carbapenemases in a single strain confers a higher level of drug resistance and further complicates clinical management (Jia et al., 2022). Moreover, NDM and IMP are often carried by mobile genetic elements such as plasmids or transposons, which facilitates the horizontal transfer of these resistance genes among different Enterobacteriaceae species (Bush and Bradford, 2019). To date, *R. ornithinolytica* producing either *bla*_IMP–4_ or *bla*_NDM–1_ has been reported (Yu et al., 2020; Zou et al., 2020; Zhu et al., 2023). However, to our knowledge, no reports have described *R. ornithinolytica* carrying both *bla*_IMP–4_ and *bla*_NDM–1_ on the same plasmid. In this study, we identified a clinical strain of *R. ornithinolytica* (designated he2023) that carries an IncHI5 plasmid co-harboring the *bla*_NDM–1_ and *bla*_IMP–4_ genes; we present its detailed structural characterisation here for the first time.

## Materials and methods

### Bacterial strains

Strain he2023 was isolated in June 2023 from a sputum specimen obtained from a 75-year-old patient with lymphoma in the Department of Hematology, Affiliated Cancer Hospital of Zhengzhou University. The patient subsequently developed pneumonia and ultimately died of septic shock. This study received ethical approval from the Bioethics Committee of Zhengzhou University Affiliated Cancer Hospital and Henan Cancer Hospital (Ethical Review Number: 2024-key-0124-001).

### Antimicrobial susceptibility testing

The BD Phoenix-M50 fully automated bacterial identification and antimicrobial susceptibility testing (AST) system was used to identify strain he2023 and to perform susceptibility testing. Matrix-assisted laser desorption/ionization time-of-flight (MALDI-TOF) mass spectrometry was used for confirmatory identification. The Minimum Inhibitory Concentrations (MICs) to ceftazidime/avibactam and tigecycline were determined using E-Test Strips (Liofilchem, Roseto degli Abruzzi, Italy). Antimicrobial susceptibility interpretation followed by the Clinical and Laboratory Standards Institute [CLSI] (2023) guidelines for all agents, except polymyxin and Tigecycline, for which the 2019 European Committee on Antimicrobial Susceptibility Testing (EUCAST) criteria were used. The susceptibility results for imipenem and meropenem were further verified using E-test (Liofilchem, Roseto degli Abruzzi, Italy). *Escherichia coli* ATCC25922 was served as a quality control strain in the susceptibility testing assay.

Carbapenemase production in he2023 was screened using the NG-Test^®^ CARBA 5 (Zhongshengzhongjie, Changsha, China).

### Conjugation experiments and plasmid stability

For conjugation assay, strain he2023 (donor) and *E. coli* J53 (recipient) were grown to logarithmic phase; equal volumes (1 mL each) were inoculated into 100 mL nutrient broth, mixed and incubated statically overnight at 37 °C. After incubation, 50 μl aliquots were plated onto LB agar containing 2 μg/ml meropenem and 100 μg/ml sodium azide. After overnight incubation, presumptive transconjugants were identified by the method described above. Plasmids from the donor and any transconjugants were analyzed by 0.5% agarose gel electrophoresis. Plasmid bands were gel-extracted and subjected to PCR to detect carbapenemase genes. The oriTfinder website^1^ was used to conduct a detailed analysis of the conjugation module in order to determine whether the plasmid is conjugative.

The plasmid-carrying strain he2023 was continuously subcultured at 37 °C (1:1000) for 10 days to assess plasmid stability. On the 10th day, the culture was serially diluted and plated on LB plates with or without meropenem (1 mg/L). The plasmid retention rate was calculated by dividing the number of colonies on LB plates supplemented with meropenem by the number of colonies on LB plates. Multiplex PCR was then performed to detect *bla*_NDM–1_ and *bla*_IMP–4_ genes.

### Whole-genome sequencing

Whole-genome sequencing of strain he2023 was performed using Illumina NovaSeq 6000 and Oxford Nanopore MinION platforms, and reads were combined for hybrid genome assembly. Plasmid sequences containing antimicrobial-resistance genes were submitted to GenBank^2^ to obtain sequence numbers. Antimicrobial resistance genes and plasmid replicons were identified using ResFinder 4.1 and PlasmidFinder 1.3^3^. Virulence genes were identified using the virulencefactor database (VFDB), and transposon and insertion sequence (IS) elements were using the ISFinder database^4^. Comparative genomic circular maps and synteny analyses were generated with BRIG v0.95 and Easyfig 2.2.5, respectively, to analyze structural variation among related plasmids.

## Results

### Isolate characteristics and susceptibility testing

Strain he2023 was identified as *Klebsiella pneumoniae* by the BDPhenix-M50 system, but both MALDI-TOF and WGS identified the isolate as *R. ornithinolytica*. The isolate was susceptible to amikacin, Tigecycline, and polymyxin B, and resistant to gentamicin, cefazolin, cefotaxime, ceftazidime, cefepime, aztreonam, ampicillin, amoxicillin-clavulanic acid, ampicillin-sulbactam, piperacillin-tazobactam, chloramphenicol, levofloxacin, trimethoprim-sulfamethoxazole, Aztreonam, imipenem, and meropenem (Table 1). The MIC for imipenem was >8 μg/mL, and for meropenem was 8 μg/mL. Results of the NG-Test^®^ CARBA 5 indicated that isolate he2023 harbored *bla*_NDM–1_ and *bla*_IMP–4_ genes.

**TABLE 1:** Antimicrobial susceptibility profiles of he2023 against different antimicrobials.

Strains	he2023																
Antimicrobials agents	MEM	IMP	TZP	SXT	PB	CAZ	FEP	CRO	LVX	GEN	SAM	CHL	CIP	AMK	CZA	ATM	TGC
MIC (μ/mL)	8	>8	>64/4	>4/76	≤0.5	>16	>16	>32	8	>8	>16/8	>16	>4	≤8	≥128/4	16	2
Results	R	R	R	R	S	R	R	R	R	R	R	R	R	S	R	R	S

MEM, meropenem; IMP, imipenem; TZP, piperacillin/tazobactam; SXT, compound sulfamethoxazole; PB, polymyxin B; CAZ, ceftazidime; FEP, cefepime; CRO, ceftriaxone; LVX, levofloxacin; GEN, gentamicin; SAM, ampicillin/sulbactam; CHL, chloramphenicol; CIP, ciprofloxacin; AMK, amikacin; TGC, tigecycline; CZA, ceftazidime/avibactam; ATM, aztreonam; TGC, Tigecycline; R, resistant; S, sensitive.

### Conjugation experiments and plasmid stability

Multiple mating attempts to transfer plasmid from *R. ornithinolytica* He2023 to *E. coli* J53 were unsuccessful. Under the conditions tested, the plasmid appeared non-transferable. The plasmid’s conjugation-related functional regions were predicted using oriTfinder (see text footnote 5). The analysis indicated that plasmid pNDM-IMP encodes a Type IV secretion system (T4SS) but lacks an identifiable origin of transfer (oriT), relaxase, and T4CP (type IV coupling protein).

The stability of plasmid pNDM-IMP was also evaluated during serial passage in the laboratory last for 10days. It displayed high stability, as the retention rates were still over 92% at the end of the experiment for both *bla*_NDM–1_ and *bla*_IMP–4_.

### Genomic characterisation analysis of strain he2023

Whole-genome sequencing revealed that he2023 comprises one chromosome and two plasmids (p2 and pNDM-IMP). The chromosome (5,624,232 bp; GC content of 56.0%) harbors several resistance determinants, including the multidrug efflux pump genes *oqxB* and *oqxA*, β-lactamase resistance gene *bla*_PLA1a_, and the fosfomycin resistance gene *fosA*. Furthermore, the chromosomes of strain he2023 carried a large number of virulenceassociated factors, such as type 1 and type 3 fimbriae, capsule, iron uptake (Ent siderophore and yersiniabactin), ferric aerobactin receptor protein (*iutA*), type 6 secretion systems (*T6SS-I*, *T6SS-II*, *T6SS-III*) and lipopolysaccharide (*LPS*) biosynthetic locus (*rfb*). No virulence genes were discovered on two plasmids. Plasmid p2 (92,321 bp; GC content of 46.57%) carries multiple resistance genes including the quinolone resistance gene *qnrS1*, β-lactamase resistance genes *bla*_CTX–M–3_ and *bla*_TEM–1B_; the phenicol resistance gene *floR*; tetracycline resistance gene *tet(A)*; macrolide resistance genes *mph(A)* and *msr(E)*, sulfonamide resistance genes *sul1* and *sul2*, the trimethoprim resistance gene *dfrA27*, the rifampin resistance gene *arr-3*; and aminoglycoside resistance determinants (*aac(6’)-Ib-cr, aac(3)-IId, aph(3’)-Ia, aph(6)-Id, aph(3”)-Ib, aadA16* as well as *qacE*. Plasmid pNDM-IMP (23,8021 bp; GC content of 52.92%) and contains 10 resistance determinants. These include tetracycline resistance gene *tet(D)*, macrolide resistance genes *mph(E)*, and *msr(E)*; sulfonamide resistance gene *sul1*, the rifampin resistance gene *arr-3*, the aminoglycoside resistance gene *aph(3’)-VIa*; *qacE*; a bleomycin resistance gene *ble*_MBL_; and the carbapenemase genes *bla*_IMP–4_ and *bla*_NDM–1_. Among these genes, the carbapenem resistance genes *bla*_NDM–1_ and *bla*_IMP–4_ are of particular importance. The details are shown in Table 2.

**TABLE 2:** Whole genome information for he2023.

Name	Genome size (bp)	GC (%)	Inc	Drug resistance genes	Accession number
chromosome	5624232	56.00%	–	*OqxB; OqxA; blaPLA1a; fosA*	CP139090.1
pNDM-IMP	238021	46.57%	IncHI5	*tet(D); mph(E); msr(E); sul1; aph(3’)-VI; sul1;qacE;arr-3;ble(MBL)*;*bla*_IMP–4_;*bla*_NDM–1_	CP139091.1
p2	92321	52.92%	IncFII(K)/IncQ1	*qnrS1*; *bla*_CTX–M–3_; *bla*_TEM–1B_; *floR*; t*et(A); mph(A); qacE;dfrA27; arr-3; aac(6’)-Ib-cr; aac(3)-IId;aph(3’)-Ia;mph(A);sul1;aadA16;* *aph(6)-Id; aph(3”)-Ib; sul2*	CP139092.1

### Structural characterization of the IncHI5 plasmid pNDM-IMP Co-harboring *bla*_*NDM–*1_ and *bla*_IMP–4_

**FIGURE 1:**
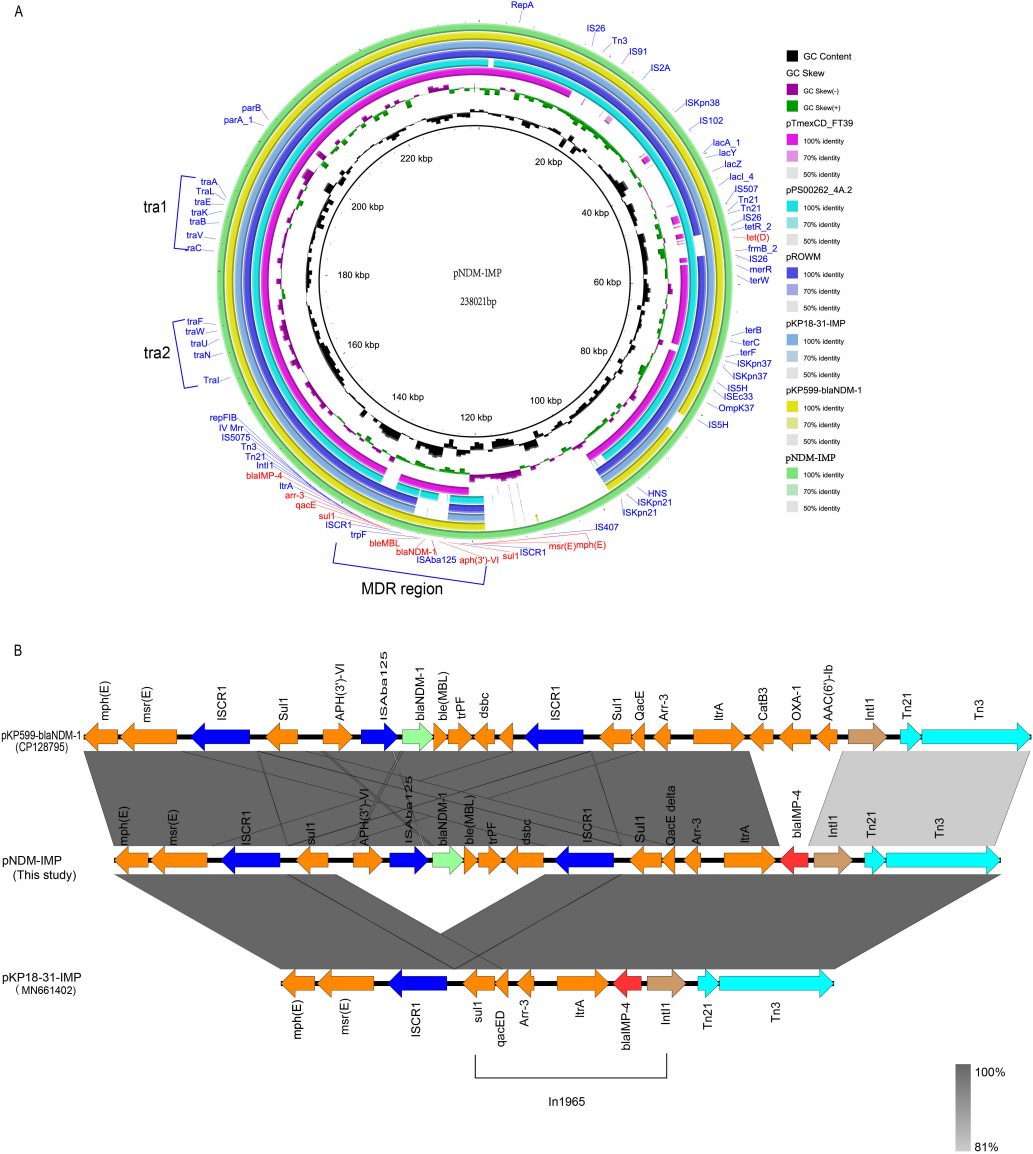
**(A)** Shows a circular map of plasmid pNDM-IMP, highlighting gene regions, repA, bla IMP, bla NDM, and MDR sections with color-coded rings denoting sequence identities. **(B)** Displays a linear alignment of three plasmids, pKP99-*bla*_NDM1_, pNDM-IMP, and pKP18-31-MP, illustrating gene synteny and shared regions with shaded areas representing homology levels.

The results indicate that the pNDM-IMP plasmid is the key plasmid responsible for the multidrug resistance in this strain. Plasmid pNDM-IMP is a circular molecule of 238,021 bp and encodes approximately 350 open reading frames (ORFs). Plasmid pNDM-IMP was belonged to IncHI5,because it contained the replicon combination *repHI5B* and *repFIB* unique to IncHI5 plasmids (Soulier et al., 1988). BLAST comparisons showed high sequence identity with several known plasmids: pNDM-IMP shared 74% query coverage and 99.98% identity with plasmid pTmexCD_FT39 (GenBank accession no. CP132737), 88% query coverage and 99.92% identity with pPS00262_4A.2 (GenBank accession no. CP127498) from *Klebsiella aerogenes*, 89% query coverage and 99.99% identity with pKP18-31-IMP (GenBank MN661402) from *Klebsiella quasipneumoniae* strain KP18-31 (Zhengzhou, Henan, China), 88% query coverage and 99.77% identity with unnamed plasmid pROWM (GenBank MT136604) from *R.ornithinolytica* ROWM (Tianjin, China), and 90% query coverage and 99.99% identity with pKP599-blaNDM-1(GenBank CP128795) from *K. aerogenes* KP599 (Hangzhou, China) (Figure 1A). Comparative analysis indicated pNDM-IMP and related plasmid share a conserved backbone. This backbone includes dual replicons (*repHI5B* and *repFIB*), a partitioning gene cluster (*parA* and *parB)*, two conjugation (*tra*) regions, and a multidrug-resistance (MDR) region harboring multiple antimicrobial resistance genes. Notably, although the backbone is conserved, the specific resistance genes present in the MDR region vary among these plasmids.

Plasmids pKP18-31-IMP and pKP599-blaNDM-1, which exhibited high sequence coverage and identity with plasmid pNDM-IMP, were selected for collinearity analysis of their multidrug resistance (MDR) regions (Figure 1B). The MDR region of pNDM-IMP is 14 kb and contains multiple resistance genes determinants, including *mph(E)*, *msr(E)*, *aph(3’)-VIa*, *ble*_MBL_, *sul1*, *arr-3*, as well as the carbapenem resistance genes *bla*_NDM–1_ and *bla*_IMP–4_. In contrast, pKP18-31-IMP carries only *bla*_IMP–4_, which shares an identical sequence context with the corresponding region in pNDM-IMP. Both *bla*_IMP–4_ loci are embedded within a class 1 integron. However, pKP18-31-IMP lacks *aph(3’)-VIa*, *ble*_MBL_ and *bla*_NDM–1_. Compared with pNDM-IMP, pKP599-blaNDM-1 has an almost identical MDR region and also carries *bla*_NDM–1_; however, the region corresponding to *bla*_IMP–4_ in pNDM-IMP is replaced in pKP599 by *catB3*, *bla*_OXA–1_, and *aac(6’)-Ib*.

**FIGURE 2:**
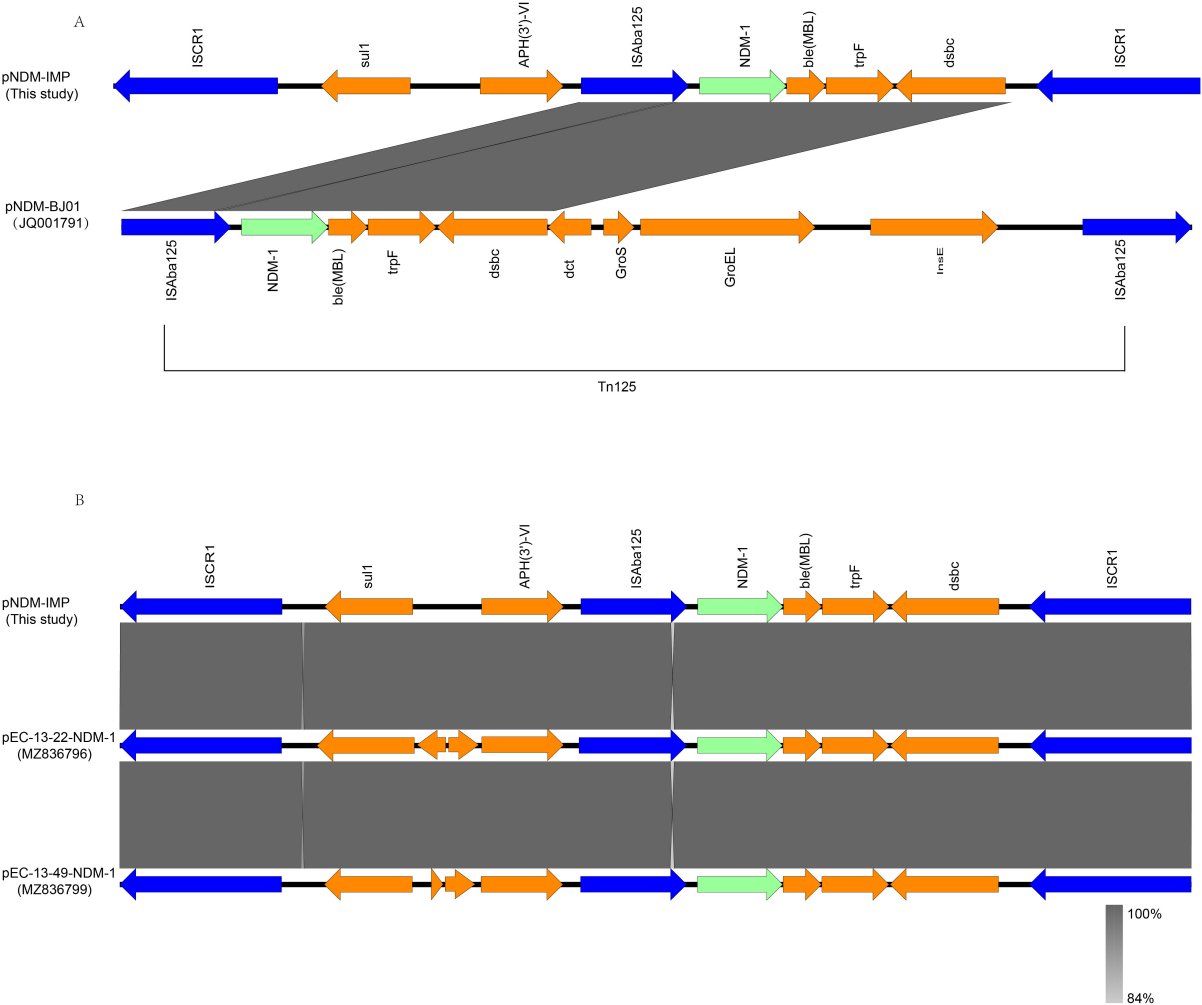
Two synteny diagrams comparing genetic structures. **(A)** Shows a comparison between pNDM-IMP (this study) and pNDM-BJ01 with overlapping regions highlighted. Genes are depicted with colored arrows. **(B)** Compares pNDM-IMP with pEC-13-22-NDM-1 and pEC-13-49-NDM-1, showing shared regions shaded in gray. The legend indicates similarity percentages in grayscale.

The genetic contexts flanking *bla*_NDM–1_ and *bla*_IMP–4_ were analyzed using Easyfig 2.3. The analyses revealed that *bla*_NDM–1_ is preceded upstream by insertion sequence *ISAba125*, and its downstream region contains *ble*_MBL_. The *bla*_NDM–1_ gene is located within the structure *ISAba125-bla_*NDM–1*_-ble*_MBL_*-trpF-dsbC*, which shows a relatively high identity to the corresponding region of transposon *Tn125* (Figure 2A). Furthermore, this region is flanked by two copies of *ISCR1* (insertion sequence common region 1), forming a highly conserved structure: ISCR1-*sul-aph(3’)-VI-ISAba125-bla*_NDM–1_-*ble*_MBL_-*trpF-dsbC-ISCR1* (Figure 2B). The gene *bla*_IMP–4_ is located downstream of *bla*_NDM–1_ within a novel class 1 integron designated *In1965*; this integron also contains a reverse transcriptase (*ltrA*; *K1.pn.I3*). The integron structure is *sul1-qacE-arr-3-ltrA(K1.pn.I3)*-*bla*_IMP–4_-*IntI1.* This region is identical to the corresponding region in pKP18-31-IMP. Downstream of the integron, remnants of *Tn21* and a *Tn3* transposase were identified.

## Discussion

With the increasing use of novel β-lactamase inhibitors, the detection rate of class B metallo-β-lactamases (MBLs) has risen significantly, posing a serious challenge to the global treatment of carbapenem-resistant Enterobacteriaceae (CRE) infections (Sader et al., 2023). NDM and IMP are typical members of MBLs, with NDM-1 first identified in *K. pneumoniae* in India in 2008 (Yong et al., 2009). Currently, NDM-1 is widely present in various drug-resistant bacterial strains. The increasing prevalence of NDM-producing pathogens has seriously compromised the efficacy of carbapenems in clinical settings, and it poses a great threat to public health. According to reports, the increasing prevalence of CRE strains in China is primarily attributed to the widespread dissemination of conserved mobile genetic elements carrying the *bla*_NDM_ gene (Zhang et al., 2017). IMP-type MBLs were previously reported to be relatively uncommon among Enterobacteriaceae in China (Han et al., 2020); however, the prevalence of IMP-4 has been rising in recent years and has reached a level that warrants enhanced surveillance (Zheng et al., 2023). Because of the high heterogeneity of MBLs and their rapid dissemination among pathogens, no effective inhibitors are currently available for clinical use. The MBLs *bla*_NDM–1_ and *bla*_IMP–4_ have emerged as significant public health concerns, posing new challenges to clinical anti-infective therapy (Jia et al., 2022). In this study, we identified a strain of *R. ornithinolytica* he2023 harboring an IncHI5 plasmid that carries both *bla*_NDM–1_ and *bla*_IMP–4_ resistance genes, and we report for the first time its detailed structural characteristics.

Polymerase chain reaction results indicated that strain he2023 co-harbored the antimicrobial resistance genes *bla*_NDM–1_ and *bla*_IMP–4_, and WGS revealed that these two resistance genes are located on a 238,021-bp IncHI5 plasmid designated as pNDM-IMP. Plasmid stability testing demonstrated that pNDM-IMP could be stably maintained even in the absence of antimicrobial selective pressure, indicating that it is a highly stable plasmid and that the antibiotic resistance genes it carries can persist effectively. The plasmid-mediated spread of carbapenem resistance genes poses a serious global health threat. The *bla*_NDM_ gene has been identified in various bacterial species and is often located on plasmids with diverse replicon types, including IncA/C, IncF, IncH, IncN, IncL/M, IncP, IncR, IncX, and IncY, as well as non-typeable plasmids (Wu et al., 2019). Among these, IncX3 plasmids are relatively common in China (Dang et al., 2020). The *bla*_IMP_ gene is typically integrated into broad-host-range conjugative plasmids and has been associated with multiple plasmid types such as A/C, L/M, HI2, N, and F (Liu W. et al., 2021). In this study, both *bla*_NDM–1_ and *bla*_IMP–4_ were found to co-exist on an IncHI5 plasmid. IncHI5 plasmids are broadly distributed among bacteria and serve as multidrug-resistant vectors capable of horizontal transfer, playing a key role in the dissemination of heavy metal and antibiotic resistance genes (Cain and Hall, 2012). In China, IncHI5 plasmids may serve as critical elements enabling the rapid dissemination of carbapenemase-encoding genes (Zhu et al., 2020). Besides *bla*_IMP_, IncHI5 plasmids have also been reported to carry *bla*_VIM_, *bla*_NDM_, *bla*_KPC_, and *bla*_SIM–1_ (Luo et al., 2022; Xie et al., 2025). Several studies further identified IncHI5 plasmids as important vectors for *bla*_NDM_, emphasizing their critical role in resistance gene dissemination (Liu Z. et al., 2021). It has also been reported that 52.5% of *bla*_IMP_ carrying plasmids belong to the IncHI5 type, suggesting that IncHI5 may have become a major vehicle for the spread of *bla*_IMP_ (Liu et al., 2024). Therefore, the co-occurrence of *bla*_NDM–1_ and *bla*_IMP–4_ on an IncHI5 plasmid in a clinical strain of *R.ornithinolytica* observed in this study is unlikely to be a random event.

Plasmid pNDM-IMP and related plasmids shared nearly identical backbone structures, with the most notable variation occurring in the multidrug resistance region. Around the resistance genes, several mobile genetic elements were found, such as integrons, transposons, and insertion sequences. In addition to the conjugative plasmids, thecapture, accumulation, and dissemination of resistance genes are largely due to the actions of mobile genetic elements, includinginsertion sequences, transposons, gene cassettes, and integrons (Ai et al., 2021). Resistance genes can be recruited into variable genetic loci flanked by insertion sequence elements and transposons, thereby promoting their widespread dissemination (Wang et al., 2017). Thus, the transfer, insertion, deletion, and recombination of resistance genes with various mobile genetic elements likely contributed to the development of the highly chimeric multidrug resistance region in plasmid pNDM-IMP, which co-harbors *bla*_NDM–1_ and *bla*_IMP–4_. Additionally, genes associated with heavy metal resistance, such as mer and ter, were identified on the backbone of plasmid pNDM-IMP. The co-localisation of heavy metal resistance genes and antimicrobial resistance genes on the same plasmid provides a selective advantage under heavy metal pressure, which may further promote the dissemination of resistance genes (Zhu et al., 2020). We also found that pNDM-IMP exhibits high homology with pTmexCD_FT39 found in sewage, indicating a link between environmental and human reservoirs for antibiotic resistance genes.

The *bla*_NDM–1_ gene is located within a structure composed of *ISAba125*-*bla*_NDM–1_-*ble*_*MBL*_-*trpF*-*dsbc*, which exhibits high nucleotide sequence identity with the corresponding region of transposon *Tn125*. Therefore, the conserved segment containing *ISAba125*-*bla*_NDM–1_-*ble*_MBL_-*trpF*-*dsbC* is likely derived from

*Tn125* (Zhu et al., 2020). Furthermore, the structure harboring *bla*_NDM–1_ is flanked on both sides by insertion sequence *ISCR1*, forming a highly conserved structure *ISCR1*-*sul1*-*APH(3’)-VIa*-*ISAba125*-*bla*_NDM–1_-*bleMBL*-*trpF*-*ISCR1*, which is essentially identical to that observed in *Escherichia coli* plasmids pEC-13-22-NDM-1 and pEC-13-49-NDM-1 (Dong et al., 2022). *ISCR1* elements are commonly found within complex class 1 integrons and possess a strong capacity for capturing resistance genes (Toleman et al., 2006a). They play a significant role in the dissemination of resistance determinants and the recombination of multiple resistance regions, and can provide promoter sequences that enhance the expression of adjacent

Genes (Deng et al., 2009). *ISCR1* is often located adjacent to antibiotic resistance genes and facilitates the mobilization of upstream DNA through rolling-circle replication, demonstrating its potential in mediating the transfer of resistance genes (Toleman et al., 2006b). The repeated occurrence of two *ISCR1-sul1* units within this multidrug resistance region strongly suggests that at least two independent rounds of *ISCR1-*mediated replication/capture events have occurred, effectively integrating distinct resistance modules. Therefore, we speculate that *ISCR1* elements playing a crucial role in the dissemination of *bla*_NDM–1_ and other resistance genes.

The *bla*_IMP–4_ gene is frequently located within class I integrons, often found downstream of *bla*_NDM–1_. Class I integrons are common carriers of the *bla*_IMP–4_ gene, including *In1589*, *In823*, *In1310*, *In809*, and others, responsible for the dissemination of the *bla*_IMP–4_ (Huang et al., 2021; Liu W. et al., 2021). In the present study, the *bla*_IMP–4_ gene was incorporated into the IntI1 integrase, giving rise to a novel integron designated *In1965*, characterized by the genetic structure *sul1*-*qacE*-*arr-3*-*ltrA*-*bla*_IMP–4_-*IntI1*. A similar arrangement has been previously reported in *K. quasipneumoniae* pKP18-31-IMP (Dong et al., 2023). The spread of integrons has accelerated the dissemination of antibiotic resistance genes (Souque et al., 2021). Importantly, the presence of the class 1 integron downstream of *bla*_NDM–1_ allows the plasmid to harbor multiple resistance genes, thereby conferring a multidrug-resistant phenotype. The intI1 integrase plays a crucial role in facilitating the accumulation of diverse resistance determinants within plasmids (Li et al., 2024). Based on the above, we speculate that the dissemination of *bla*_IMP–4_ is associated with the structure of class 1 integrons, and *ISCR1* may have facilitated the integration of the *bla*_NDM–1_ gene into the IncHI5 plasmid.

Tests assessing the self-transferability of this resistant plasmid indicated that pNDM-IMP could not be transferred into the recipient strain *J53*, suggesting that it is a non-mobilisable plasmid. Although the pNDM-IMP plasmid is non-transferable, the abundance of resistance genes, the presence of mobile genetic elements that facilitate exchange, additional non-carbapenem resistance determinants, and co-selective pressure from heavy metal resistance genes may still enable the persistence and dissemination of the *bla*_NDM–1_ and *bla*_IMP–4_ to other bacteria. Therefore, this seemingly “non-transferable” plasmid may be a potential reservoir of drug resistance genes, and it is crucial to enhance the monitoring of such positive plasmids.

## Conclusion

In summary, this study reports for the first time that the carbapenem resistance genes *bla*_NDM–1_ and *bla*_IMP–4_ of *R. ornithinolytica* are simultaneously located on the non-transferable IncHI5 plasmid. This super resistance gene cluster is composed of multiple mobile elements working together. This emphasizes that in clinical microbiological monitoring and drug resistance prevention and control, in addition to focusing on the transferability of plasmids, it is even more necessary to deeply analyze the mobile genetic elements they carry.

## Data Availability

The datasets presented in this study can be found in online repositories. The names of the repository/repositories and accession number(s) can be found below: https://www.ncbi.nlm.nih.gov/genbank/, CP139090-CP139092.

## Footnotes

1
https://bioinfo-mml.sjtu.edu.cn/oriTfinder/

2
https://www.ncbi.nlm.nih.gov/genbank/

3
http://www.genomicepidemiology.org/

4
https://www-is.biotoul.fr/
